# Editorial: Photosynthesis under abiotic stress

**DOI:** 10.3389/fpls.2025.1732692

**Published:** 2025-11-18

**Authors:** Michael Moustakas, Anelia Dobrikova, Alexander G. Ivanov

**Affiliations:** 1 Department of Botany, School of Biology, Aristotle University of Thessaloniki, Thessaloniki, Greece; 2 Institute of Biophysics and Biomedical Engineering, Bulgarian Academy of Sciences, Sofia, Bulgaria; 3 Department of Biology, University of Western Ontario, London, ON, Canada

**Keywords:** drought stress, salt stress, light stress, temperature stress, nutrient deficiency, formaldehyde, reactive oxygen species, CO_2_ concentration

## Photosynthesis under abiotic stress

Abiotic stress factors such as drought, salinity, extreme temperatures, UV radiation, high light, nutrient deficiency are the main reasons for the reduction of crop yields and food production worldwide (Kopecká et al., 2023; Moustakas, 2025). Photosynthesis is the device of crop productivity, but likewise, it is a complex process that is extremely responsive to various abiotic stresses with a multifaceted relationship to the growth and productivity of plants and aquatic photosynthetic organisms, such as algae and cyanobacteria (Gururani et al., 2015). As a result of drought stress, for example, remarkable changes in growth, photosynthesis, enzymatic activities, and biomass production, occur (Croce et al., 2024). In plants, the decreased photosynthetic efficiency, which is linked to both stomatal and non-stomatal limitations, is the result of a disruption of either biochemical or/and photochemical activity and increased oxidative damage by the surplus reactive oxygen species (ROS) accumulation, which can harm the chloroplast, and particularly photosystem II (PSII) (Moustakas et al., 2022b). However, plants have developed several effective approaches at the morphological, physiological, and biochemical levels, allowing them to avoid and/or tolerate drought stress (Moustakas, 2025).

Photosynthesis of food crops under environmental stress conditions has been considered to be a real challenge for scientists and crop breeders in order to fulfil the huge demand for food in the world (Morales et al., 2020; Croce et al., 2024). The fast progress of synthetic biology tools now offers new scenarios towards totally new designs of improved photosynthetic systems and adjusting photosynthesis to the increasing demands of our changing climate (Zhu et al., 2022; Croce et al., 2024). Photosynthetic manipulation offers new prospects for enhancing crop yield (Zhu et al., 2022). Therefore, detailed information on photosynthetic organism responses and a better understanding of the photosynthetic machinery to environmental stresses could help in developing new crops with higher yields (Muhammad et al., 2021). Manipulating photosynthetic organisms with enhanced abiotic stress tolerance will involve a complete understanding of ROS signaling and the regulatory functions of several other components, including secondary metabolites, transcription factors, phytohormones, and protein kinases, in the responses of photosynthetic apparatus to abiotic stress (Gururani et al., 2015).

To meet global food and feed requirements, considering current climate change scenarios, it is essential to recognize how photosynthetic organisms respond and adapt their metabolism to abiotic stress (Zhu et al., 2022; Leister, 2023; Wani, 2023; Croce et al., 2024). Thus we must understand better (i) the way photosynthetic machinery is working, and how it can be further enhanced, (ii) elucidate the mechanisms of the photosynthetic responses to abiotic stress and thus contribute to a better understanding of photosynthesis in plants and aquatic photosynthetic organisms under stress that can help in the development of realistic interventions for increasing agricultural productivity, (iii) Detect the steps or mechanisms where photosynthetic systems are suboptimal under different environmental conditions, and then optimizing these steps for best performance, which represents a key research target in present photosynthetic improvement efforts to increase the ability of crops to face climate change that influence crop production detrimentally.

In this editorial article, we summarize the articles in this Research Topic that update the readers on the subject and can be useful for scientists working on this Research Topic, since recent advances in the subject were attractively presented.

Global crop production faces rising hazards from the increased frequency, intensity and duration, of drought stress incidents owing to climate change, and its effects when combined with other stress becomes more noticeable (Moustaka et al., 2025). Chlorophyll fluorescence analysis has been commonly used to evaluate photosynthetic function and to assess plant tolerance to different environmental stresses (Moustakas et al., 2022a). Naseer et al. by using chlorophyll fluorescence analysis revealed that both reduced irrigation and increased shading durations negatively impact winter wheat during the grain-filling stage, with interactive effects causing the most severe damage to physiological functions and leading to significant yield reductions. However, the combined effects of abiotic stresses do not always outcome in a simple additive response, but rather, they may produce complex and interconnected physiological and molecular mechanisms (Moustaka et al., 2025). This was the case in a simulation study on sweet sorghum under varying temperature and CO_2_ conditions which revealed that elevated CO_2_ concentrations improve photosynthesis and water use efficiency, but this advantage is reduced at higher temperatures (Yang et al.). The conclusion of this study was that while elevated CO_2_ alone can lead to stomatal closure increasing water use efficiency, this consequence is diminished when combined with heat stress, highlighting the complex interplay between these factors.

Li et al. observed that formaldehyde, a common gaseous pollutant emitted by buildings and decorative materials, causes structural damage, reduces pigment content, decreases photosynthetic efficiency, and increases ROS production in the moss *Racomitrium japonicum*. Furthermore, they noticed that different formaldehyde concentrations trigger distinct stress-response pathways in *R. japonicum*, with the low and moderate formaldehyde concentrations (< 50 mg/m^3^) activating the antioxidant enzymic system to mitigate ROS accumulation but with the high-concentration (100 mg/m^3^) to suppress the antioxidant enzymic activity. The study suggested that an effective indoor formaldehyde monitoring system is essential.

The dramatic decrease in atmospheric CO_2_ concentration during Oligocene was directly linked to evolution of C4-type photosynthesis (Ehleringer et al., 1991). Miao et al. reported that under low CO_2_ conditions, C3 plants like *Arabidopsis thaliana* refurbish their metabolism to recycle ammonium by increasing the expression of most genes encoding the C4-related enzymes and transporters, genes that are involved in photorespiration, and genes that are involved in ammonium refixation. They proposed an “evolutionary hitchhiking” process, where the necessary metabolic adjustments for ammonium recycling under low CO_2_ conditions co-opted the expression of C4-related genes that were already present in the C3 genome.

Soil salinization is one of the main constraints to crop production in arid and semi-arid regions. Zhou et al. explore the efficacy of combined application of organic and inorganic nitrogen as a valuable strategy to improve the productivity of maize in saline soils. Through series of carefully planned photosynthetic and biochemical experiments the authors concluded that organic and inorganic nitrogen application mitigates salinity stress effects on maize. In mildly saline soils, inorganic nitrogen application (U1) and organic nitrogen replacing 50% (O1), was optimal and improved yield primarily through enhanced photosynthetic performance, whereas in moderately saline soils O1 nitrogen application was optimal and yield formation was driven by an integrated influence of growth traits, photosynthetic parameters, and catalase (CAT) activity. The results provide insights and better understanding of nitrogen forms management in improving crop productivity in saline environments.

The effects of nitrogen as the most essential and key limiting nutrient factor for plant growth and overall plant development were also studied by Li et al. using varying nitrogen levels on photosynthetic performance and photosynthetic nitrogen use efficiency (PNUE) in two tea cultivars. The presented results demonstrate that increasing nitrogen levels can significantly enhance the photosynthetic capacity of tea plants and improve photosynthetic nitrogen use efficiency up to an optimal level, after which excessive nitrogen can reduce these benefits by decreasing nitrogen allocation to photosynthetic structures, causing a decline in net photosynthetic rate and photosynthetic nitrogen use efficiency. Thus, optimizing nitrogen distribution by increasing the nitrogen content in the carboxylation and electron transport systems is expected to enhance both net photosynthetic rate and photosynthetic nitrogen use efficiency of tea plants (Li et al.).

The survival strategy of *Carex parva* and *Carex scabrirostris*, which have a competitive advantage over other turfgrass species exposed to low-light conditions was studied by (Liu et al.). The biochemical, physiological and photosynthetic experimental data reported in this study significantly advanced our understanding of the response mechanisms of *C. parva* and *C. scabrirostris* to low-light conditions. Although photosynthetic parameters, leaf physiological indicators, and biomass allocation of the two *Carex* species were significantly affected, both species demonstrated significant shade tolerance under simulated low-light environments. However, in terms of response strategies *C. scabrirostris* performs as a high photosynthesis investing species with high productivity and greater potential for application under low-light conditions compared to *C. parva* (Liu et al.).

The above studies clearly show that photosynthesis under environmental stress conditions is a real challenge for scientists that must find methods to decrease the harmful impacts on crop productivity.
